# Supplementary material for: Transcriptome Analysis Reveals Mycelial and Fruiting Responses to Lithium Chloride in Coprinopsis cinerea
Source: J Fungi (Basel). 2024 Feb 9;10(2):140. doi: 10.3390/jof10020140 (PMC10890143; doi:10.3390/jof10020140)
Supplement: Supplementary file 1 [file jof-10-00140-s001.zip › Table S1.pdf]

Table S1. Primers designed for validation of gene expression profile in *C. cinerea* transcriptome data

Primer ID	Primer sequences (5' to 3')	Amplicon size
CC1G_07486FP	AGACGCGACGGACTACTTTC	101bp
CC1G_07486RP	TAGTGCCCTGGGTTCATACG	
CC1G_04403FP	AAGGTGGTCTTTGCCCACAA	99bp
CC1G_04403RP	CCACCACGATCAACTGACGA	
CC1G_09477FP	ATGTCGCGACGGATTCCTAC	99bp
CC1G_09477RP	ATTCGGGACCAGGAGATGGA	
CC1G_00058FP	TCCTGCTACGTGGCAAATGA	100bp
CC1G_00058RP	TGGATCATCGACGCATGGTT	
CC1G_00407FP	TTCAGTCACGCGTAGGGAAG	123bp
CC1G_00407RP	AACAGTTCTCCGCTTGTCCC	
CC1G_15600FP	CCTGGCAGTGTTGAAGACCC	161bp
CC1G_15600RP	CCGATGAGACCATGCAGAAGA	
CC1G_10354FP	TCGTTGATCACGTCAGTGGAA	144bp
CC1G_10354RP	ACGGGTTACGGATGGAGACT	
CC1G_09588FP	TTAGGCGCGTGCCATTCAAG	105bp
CC1G_09588RP	TATCCCTGAGCGAACGACAC	
CC1G_00620FP	CCATTGACCGACGACCCTAC	96bp
CC1G_00620RP	TACGCCGATGGAGACACAGA	
CC1G_01088FP	AGCGCTGAGGAAGCAGAAAA	101bp
CC1G_0108RFP	GCTCGTCGTCTGGATCACTT	
Beta-tubulin_FP	GAGCAGTCCTGGTTGACCTC	130bp
Beta-tubulin_RP	TGTCCTTTGGCCCAGTTGTT	

FP: forward primer; RP: reverse primer.
