# Supplementary material for: Transcriptome Analysis Reveals Mycelial and Fruiting Responses to Lithium Chloride in Coprinopsis cinerea
Source: J Fungi (Basel). 2024 Feb 9;10(2):140. doi: 10.3390/jof10020140 (PMC10890143; doi:10.3390/jof10020140)
Supplement: Supplementary file 1 [file jof-10-00140-s001.zip › Table S2.pdf]

	**Table S2. Unigenes used for validation of gene expression profile in *C. cinerea* transcriptome data.**				
**Sample set**	**JGI Protein ID**	**Gene ID**	**Differential expression analysis based on RNA-Seq (log2 Fold Change)**	**Validation of the DEG by qRT-PCR (log2-ΔΔC)**	
LM1/HM	473005	CC1G_07486	1.48	1.77±0.22	
	411802	CC1G_04403	-1.95	-2.29±0.49	
	542232	CC1G_09477	1.53	1.92±0.26	
	375908	CC1G_00058	-1.95	-0.98±0.24	
	397251	CC1G_00620	-1.41	-1.92±0.48	
	461885	CC1G_01088	-1.15	-2.19±1.04	
LM2/HHK	467706	CC1G_00407	-2.54	-1.60±0.45	
	429272	CC1G_15600	4.02	3.82±0.80	
	444086	CC1G_10354	-1.38	-1.07±1.15	
	427679	CC1G_09588	-1.65	-2.59±1.82	

2-ΔΔC, relative gene expression level with beta-tubulin serving as the reference gene using qRT-PCR.Data presented as mean ± standard deviation (SD) in three replicates. HM, *C. cinerea* mycelium treated with water; LM1, *C. cinerea* treated with 70 mM LiCl; HHK, *C. cinerea* treated with water that formed hyphal knot under favorable environmental conditions for fruiting; LM2, *C. cinerea* treated with 70 mM LiCl that remained in mycelium under favorable environmental conditions for fruiting.
