# Supplementary material for: Transcriptome Analysis Reveals Mycelial and Fruiting Responses to Lithium Chloride in Coprinopsis cinerea
Source: J Fungi (Basel). 2024 Feb 9;10(2):140. doi: 10.3390/jof10020140 (PMC10890143; doi:10.3390/jof10020140)
Supplement: Supplementary file 1 [file jof-10-00140-s001.zip › Table S3.pdf]

Table S3. KEGG enrichment between different samples (p-value ≤ 0.05).

Pathway	Class	Group	p-value	FDR
**KEGG enrichment in LM1/HM-UP and LM1/HM-DOWN**				
Biosynthesis of secondary metabolites	Overview	Metabolism	6.70E-12	4.02E-10
Starch and sucrose metabolism	Carbohydrate metabolism	Metabolism	6.00E-05	0.001800386
Biosynthesis of antibiotics	Overview	Metabolism	0.000767646	0.015352915
Glycerophospholipid metabolism	Lipid metabolism	Metabolism	0.002272296	0.027543139
Tyrosine metabolism	Amino acid metabolism	Metabolism	0.002295262	0.027543139
Pyruvate metabolism	Carbohydrate metabolism	Metabolism	0.001327923	0.018171584
**KEGG enrichment in LM2/HHK-UP and LM2/HHK-DOWN**				
Steroid biosynthesis	Lipid metabolism	Metabolism	1.91E-05	0.00059773
Cell cycle - yeast	Cell growth and death	cellular processes	1.99E-05	0.00059773
DNA replication	Replication and repair	cellular processes	0.000596357	0.011927147
Meiosis - yeast	Cell growth and death	cellular processes	0.003380868	0.032617563
Mismatch repair	Replication and repair	cellular processes	0.003805382	0.032617563
Homologous recombination	Replication and repair	cellular processes	0.003805382	0.032617563
Nucleotide excision repair	Replication and repair	Genetic Information Processing	0.005998159	0.037922575
Base excision repair	Replication and repair	Genetic Information Processing	0.006466285	0.037922575
