# Supplementary material for: Transcriptome Analysis Reveals Mycelial and Fruiting Responses to Lithium Chloride in Coprinopsis cinerea
Source: J Fungi (Basel). 2024 Feb 9;10(2):140. doi: 10.3390/jof10020140 (PMC10890143; doi:10.3390/jof10020140)
Supplement: Supplementary file 1 [file jof-10-00140-s001.zip › Table S4.pdf]

**Table S4. GO enrichment between different samples (p-value ≤ 0.05).**

| | | | | | |
|---|---|---|---|---|---|
| **GO enrichment LM1/HM-UP and LM1/HM-DOWN** | | | | | |
| **ID** | **Description** | **ONTOLOGY** | **GeneRatio** | **p-value** | **FDR** |
| GO:0016705 | oxidoreductase activity | MF | 35/220 | 9.92291E-15 | 1.19564E-12 |
| GO:0020037 | heme binding | MF | 35/220 | 2.64153E-14 | 1.19564E-12 |
| GO:0046906 | tetrapyrrole binding | MF | 35/220 | 2.64153E-14 | 1.19564E-12 |
| GO:0004497 | monooxygenase activity | MF | 30/220 | 1.28384E-13 | 3.97316E-12 |
| GO:0005506 | iron ion binding | MF | 33/220 | 1.46298E-13 | 3.97316E-12 |
| GO:0048037 | cofactor binding | MF | 53/220 | 1.14396E-10 | 2.58897E-09 |
| GO:0046914 | transition metal ion binding | MF | 43/220 | 3.02542E-07 | 5.86887E-06 |
| GO:0004601 | peroxidase activity | MF | 8/220 | 1.25308E-05 | 0.000212694 |
| GO:0016684 | oxidoreductase activity, acting on peroxide as acceptor | MF | 8/220 | 3.17983E-05 | 0.000479764 |
| GO:0016831 | carboxy-lyase activity | MF | 10/230 | 1.3104E-07 | 2.31734E-05 |
| GO:0016830 | carbon-carbon lyase activity | MF | 11/230 | 1.34758E-06 | 0.000119154 |
| GO:0005975 | carbohydrate metabolic process | BP | 33/126 | 3.61855E-08 | 1.00558E-05 |
| GO:0016051 | carbohydrate biosynthetic process | BP | 9/126 | 1.93344E-07 | 2.68646E-05 |
| **GO enrichment in LM2/HHK-UP and LM2/HHK-DOWN** | | | | | |
| GO:0017062 | respiratory chain complex III assembly | BP | 6/109 | 6.07505E-07 | 9.27244E-05 |
| GO:0034551 | mitochondrial respiratory chain complex III assembly | BP | 6/109 | 6.07505E-07 | 9.27244E-05 |
| GO:0006259 | DNA metabolic process | BP | 25/108 | 2.97058E-08 | 8.41142E-06 |
| GO:0006260 | DNA replication | BP | 12/108 | 4.79964E-07 | 6.79528E-05 |
| GO:0045786 | negative regulation of cell cycle | BP | 9/108 | 1.49856E-06 | 0.000141443 |
| GO:0000075 | cell cycle checkpoint | BP | 7/108 | 2.56234E-06 | 0.000181387 |
| GO:0000819 | sister chromatid segregation | BP | 9/108 | 3.53282E-06 | 0.000200069 |
| GO:0007059 | chromosome segregation | BP | 10/108 | 5.5524E-06 | 0.000259299 |
| GO:0005975 | carbohydrate metabolic process | BP | 26/108 | 6.41019E-06 | 0.000259299 |
| GO:0007049 | cell cycle | BP | 16/108 | 8.25582E-06 | 0.000292212 |
| GO:0022402 | cell cycle process | BP | 14/108 | 9.36165E-06 | 0.000294536 |
| GO:0098813 | nuclear chromosome segregation | BP | 9/108 | 1.87495E-05 | 0.000530907 |
| GO:0051726 | regulation of cell cycle | BP | 10/108 | 2.18667E-05 | 0.000562885 |
| GO:0005618 | cell wall | CC | 12/215 | 7.45878E-07 | 2.39466E-05 |

| | | | | | |
|---|---|---|---|---|---|
| GO:0030312 | external encapsulating structure | CC | 12/215 | 7.45878E-07 | 2.39466E-05 |
| GO:0009277 | fungal-type cell wall | CC | 11/215 | 1.25152E-06 | 2.67869E-05 |
| GO:0048037 | cofactor binding | MF | 61/257 | 7.48978E-12 | 1.15106E-09 |
| GO:0005506 | iron ion binding | MF | 28/257 | 2.55923E-08 | 1.0046E-06 |
| GO:0020037 | heme binding | MF | 29/257 | 2.61471E-08 | 1.0046E-06 |
| GO:0046906 | tetrapyrrole binding | MF | 29/257 | 2.61471E-08 | 1.0046E-06 |
| GO:0016705 | oxidoreductase activity | MF | 28/257 | 5.1743E-08 | 1.59042E-06 |
| GO:0004497 | monooxygenase activity | MF | 24/257 | 1.21543E-07 | 3.11321E-06 |
| GO:0050660 | flavin adenine dinucleotide binding | MF | 22/257 | 1.8604E-07 | 4.08449E-06 |
| GO:0050662 | coenzyme binding | MF | 32/257 | 1.3018E-06 | 2.50083E-05 |
| GO:0016614 | oxidoreductase activity, acting on CH-OH group of donors | MF | 19/257 | 3.65237E-06 | 6.2368E-05 |
| GO:0005199 | structural constituent of cell wall | MF | 11/235 | 4.75879E-07 | 7.31351E-05 |
