# Supplementary material for: Transcriptome Analysis Reveals Mycelial and Fruiting Responses to Lithium Chloride in Coprinopsis cinerea
Source: J Fungi (Basel). 2024 Feb 9;10(2):140. doi: 10.3390/jof10020140 (PMC10890143; doi:10.3390/jof10020140)
Supplement: Supplementary file 1 [file jof-10-00140-s001.zip › Table S5.pdf]

Table S5. Downregulated DEGs involved in KEGG enriched pathways between LM2 and HHM.

Gene ID	JGI Protein ID	LM2 FPKM	HHK FPKM	Log2 fold change	Gene Description	Cellular processes pathway	P-value
CC1G_03033	498847	10.61	22.56	-1.09	Other/TTK protein kinase	Cell cycle - yeast	5.00E-05
CC1G_03603	471912	2.41	8.53	-1.82	Hypothetical protein		5.00E-05
CC1G_03913	460017	7.35	24.97	-1.76	Tyrosine phosphatase		5.00E-05
CC1G_05127	461884	5.77	13.75	-1.25	Rad21 protein		5.00E-05
CC1G_05320	493180	25.65	65.30	-1.35	G2/mitotic-specific cyclin cdc13		5.00E-05
CC1G_05699	448884	10.95	23.36	-1.09	Condensin complex component cnd2		5.00E-05
CC1G_12491	466770	8.86	23.65	-1.42	Nuclear condensin complex protein		5.00E-05
CC1G_13763	498107	12.86	27.30	-1.09	Hypothetical protein		5.00E-05
CC1G_13764	371091	7.59	19.34	-1.35	Smc4 chromosome structural maintenance 4-like protein		5.00E-05
CC1G_04539	182474	4.06	16.15	-1.99	Hypothetical protein	DNA replication	5.00E-05
CC1G_05722	439189	8.16	18.48	-1.18	DNA polymerase alpha catalytic subunit		5.00E-05
CC1G_09909	359386	3.88	11.45	-1.56	DNA polymerase epsilon catalytic subunit A		5.00E-05
CC1G_11474	500550	1.11	4.38	-1.98	DNA replication helicase dna2		5.00E-05
CC1G_11942	442128	8.05	19.73	-1.29	DNA primase large subunit Spp2		5.00E-05
CC1G_02333	541864	3.51	10.39	-1.57	Hypothetical protein	Homologous recombination	5.00E-05
CC1G_06567	533614	4.53	10.45	-1.21	Hypothetical protein		5.00E-05
CC1G_07448	374046	4.78	14.41	-1.59	ATP-dependent DNA helicase		5.00E-05
CC1G_07736	364090	6.07	15.87	-1.39	RAD52 DNA repair protein RADC		5.00E-05
CC1G_14217	441663	1.85	7.19	-1.96	RAD54B protein		5.00E-05
CC1G_01089	465387	1.75	4.82	-1.46	AGC/PKA protein kinase	Meiosis - yeast	0.00025
CC1G_05221	474965	4.61	20.55	-2.16	DNA mismatch repair protein MSH2	Mismatch repair	5.00E-05
CC1G_07657	538972	10.99	22.71	-1.05	DNA mismatch repair protein msh6		5.00E-05
CC1G_00769	173502	4.24	10.70	-1.34	Origin recognition complex subunit 4		5.00E-05

CC1G_09412	175000	3.84	11.70	-1.61	Cell division cycle 20 family protein	Cell cycle - yeast Meiosis - yeast	5.00E-05
CC1G_12115	439328	10.48	24.94	-1.25	WD repeat-containing protein slp1		5.00E-05
CC1G_01961	445594	6.12	23.67	-1.95	Other/WEE protein kinase		5.00E-05
CC1G_03323	393199	7.83	22.17	-1.50	Cdc6B protein		5.00E-05
CC1G_04503	449605	4.68	12.41	-1.41	Replication control protein 1		5.00E-05
CC1G_05095	373206	4.60	12.74	-1.47	Cell division control protein 45		5.00E-05
CC1G_06464	379954	1.52	3.16	-1.06	Hypothetical protein		0.00565
CC1G_07078	490518	8.29	17.83	-1.11	Other/BUB protein kinase		0.00405
CC1G_08833	354027	3.14	7.34	-1.22	Other/CDC7 protein kinase		5.00E-05
CC1G_05633	545958	26.09	58.39	-1.16	PCNA	DNA replication Mismatch repair Homologous recombination	5.00E-05
CC1G_10825	394368	6.40	15.59	-1.28	Replication factor C		5.00E-05
CC1G_00877	516390	8.63	22.49	-1.38	DNA replication factor		5.00E-05
CC1G_11689	377250	0.89	2.59	-1.54	Hypothetical protein		0.0079
CC1G_00910	493516	7.53	18.17	-1.27	Hypothetical protein		5.00E-05
