# Supplementary material for: Transcriptome Analysis Reveals Mycelial and Fruiting Responses to Lithium Chloride in Coprinopsis cinerea
Source: J Fungi (Basel). 2024 Feb 9;10(2):140. doi: 10.3390/jof10020140 (PMC10890143; doi:10.3390/jof10020140)
Supplement: Supplementary file 1 [file jof-10-00140-s001.zip › Table S6.pdf]

Table S6. The downregulated expression gene of fruiting body induction based on RNA-seq analysis.

Gene ID	JGI Protein ID	LM2 FPKM	HHK FPKM	Log2 fold change	Gene Description	KOG ID	KOG description	P-value
CC1G_06860	442684	87.06	110.00	-0.34	Phosphatidate cytidylyltransferase	KOG1440	CDP-diacylglycerol synthase	0.01085
CC1G_09235	178424	63.08	819.58	-3.70	Hypothetical protein	KOG2419	Phosphatidylserine decarboxylase	5.00E-05
CC1G_06249	361342	25.02	207.27	-3.05	Phosphatidylserine decarboxylase	KOG2419	Phosphatidylserine decarboxylase	5.00E-05
CC1G_09238	457157	1.43	38.82	-4.76	Phosphatidylserine decarboxylase	KOG2419	Phosphatidylserine decarboxylase	5.00E-05
CC1G_09240	178286	694.73	3077.27	-2.15	Phosphatidylserine decarboxylase	KOG2419	Phosphatidylserine decarboxylase	5.00E-05
CC1G_09237	468685	0.52	6.04	-3.54	Phosphatidylserine decarboxylase	KOG2419	Phosphatidylserine decarboxylase	0.0039
CC1G_06001	479579	287.96	364.45	-0.34	Inositol-3-phosphate synthase	KOG0693	Myo-inositol-1-phosphate synthase	0.0054
CC1G_15539	365798	81.62	99.83	-0.29	Hypothetical protein	KOG1649	SWI-SNF chromatin remodeling complex, Snf5 subunit	0.027
CC1G_07958	461642	2.30	4.48	-0.96	Pheromone-regulated membrane protein Prm10	KOG1245	Chromatin remodeling complex WSTF-ISWI, large subunit	5.00E-05
CC1G_07967	436798	7.56	13.92	-0.88	SNF2-family ATP dependent chromatin remodeling factor snf21	KOG0385	Chromatin remodeling complex WSTF-ISWI, small subunit	5.00E-05
CC1G_09469	440671	5.36	8.16	-0.60	Hypothetical protein	KOG1973	Chromatin remodeling protein, contains PHD Zn-finger	3.00E-04
CC1G_00100	58294	46.09	61.88	-0.43	Brg1-associatedfactor b	KOG0679	Actin-related protein - Arp4p/Act3p	0.0015
