# Supplementary figures and images for: Transcriptome Analysis Reveals Mycelial and Fruiting Responses to Lithium Chloride in Coprinopsis cinerea
Source: J Fungi (Basel). 2024 Feb 9;10(2):140. doi: 10.3390/jof10020140 (PMC10890143; doi:10.3390/jof10020140)

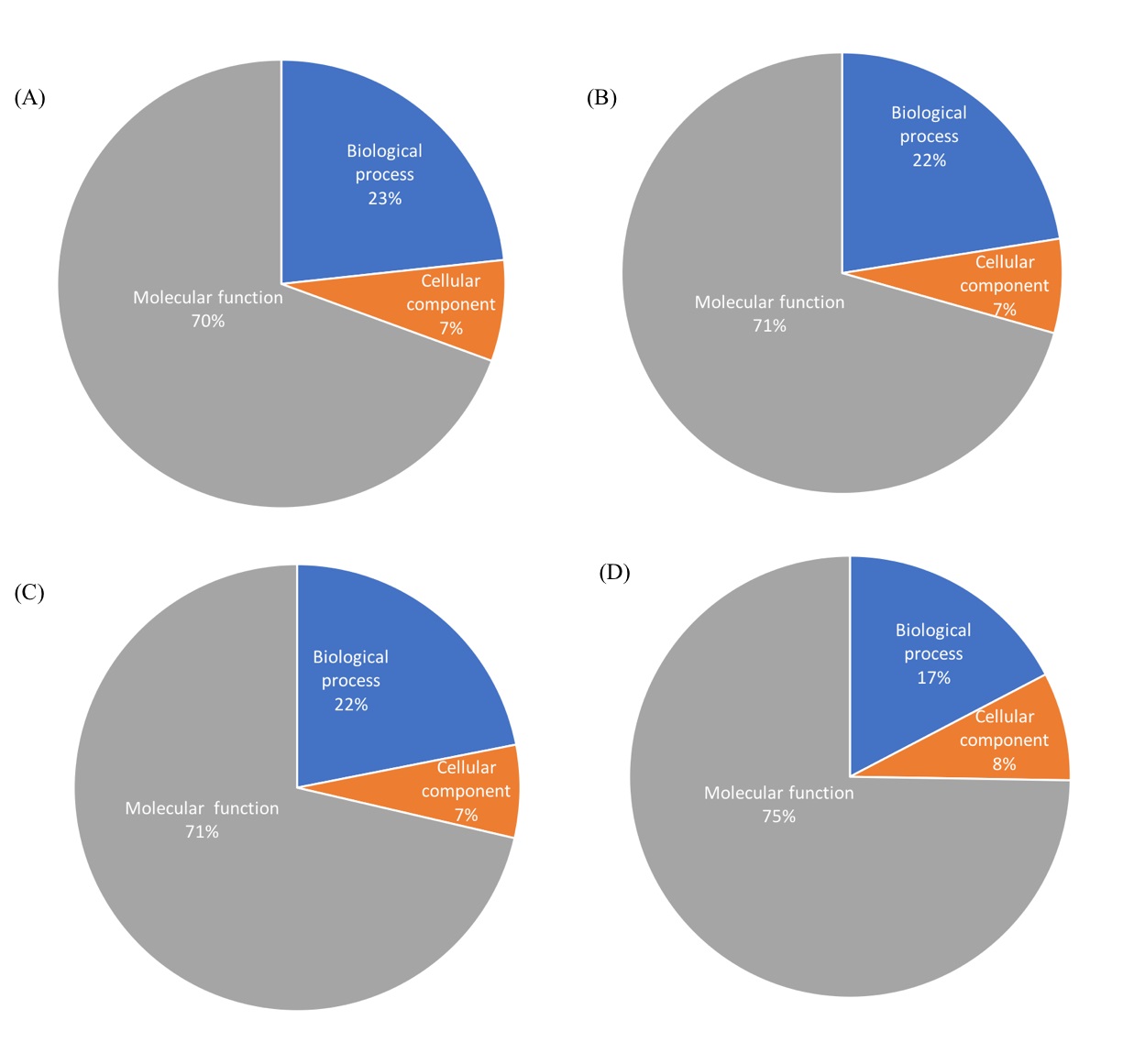

Supplement: Supplementary file 1 [file jof-10-00140-s001.zip › Figure S1.jpg]

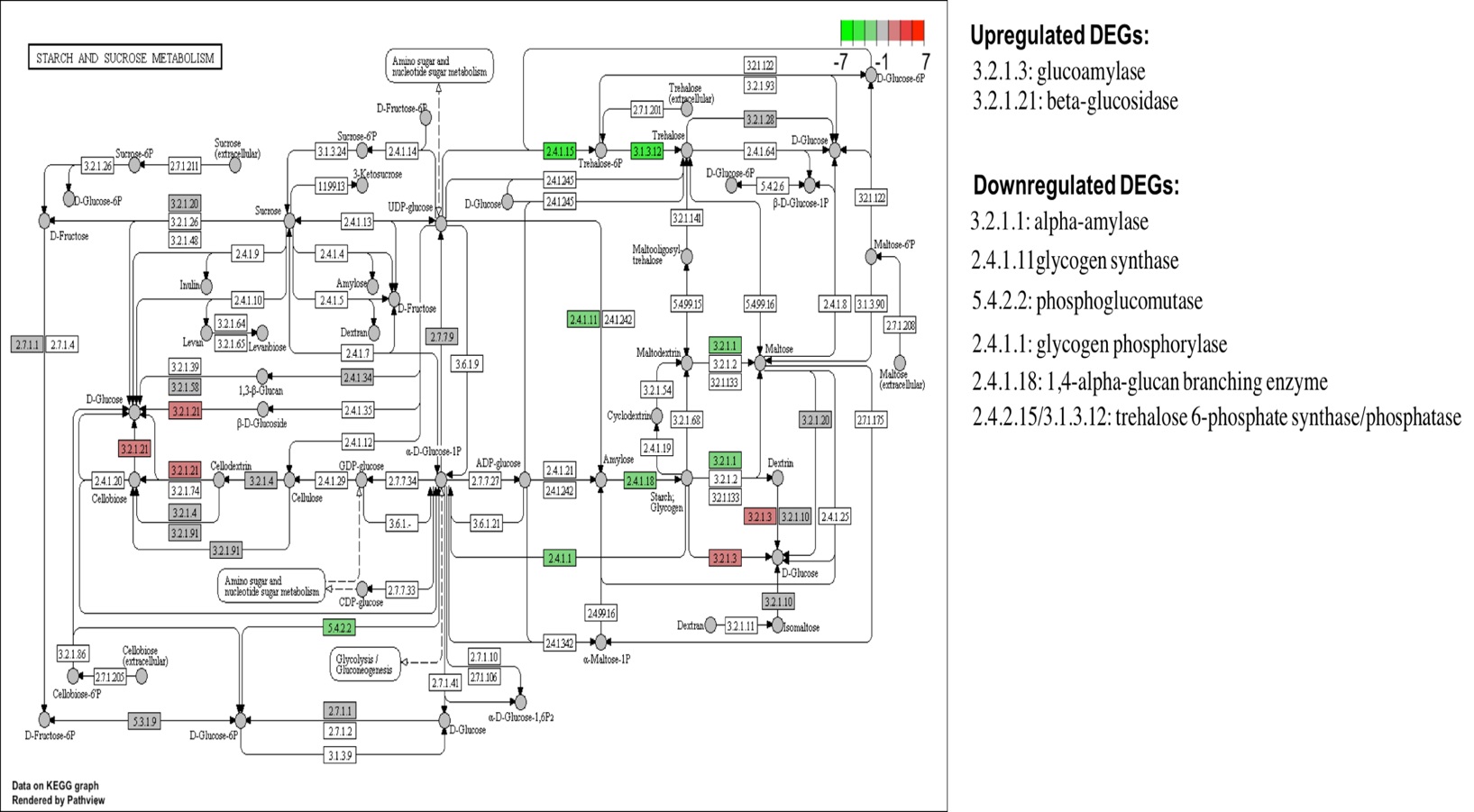

Supplement: Supplementary file 1 [file jof-10-00140-s001.zip › Figure S2.jpg]
